# A Candidate Gene Approach Identifies an *IL33 Genetic Variant* as a Novel Genetic Risk Factor for GCA

**DOI:** 10.1371/journal.pone.0113476

**Published:** 2014-11-19

**Authors:** Ana Márquez, Roser Solans, José Hernández-Rodríguez, Maria C. Cid, Santos Castañeda, Marc Ramentol, Luis Rodriguez-Rodriguez, Javier Narváez, Ricardo Blanco, Norberto Ortego-Centeno, Spanish GCA Consortium, Øyvind Palm, Andreas P. Diamantopoulos, Niko Braun, Frank Moosig, Torsten Witte, Lorenzo Beretta, Claudio Lunardi, Marco A. Cimmino, Augusto Vaglio, Carlo Salvarani, Miguel A. González-Gay, Javier Martín

**Affiliations:** 1 Instituto de Parasitología y Biomedicina López-Neyra, CSIC, Granada, Spain; 2 Systemic Autoimmune Diseases Unit, Hospital Clínico San Cecilio, Granada, Spain; 3 Department of Internal Medicine, Hospital Vall d'Hebron, Barcelona, Spain; 4 Vasculitis Research Unit, Department of Autoimmune and Systemic Diseases, Hospital Clinic, University of Barcelona, Centre de Recerca Biomèdica Cellex (IDIBAPS), Barcelona, Spain; 5 Department of Rheumatology, Hospital de la Princesa, IIS-Princesa, Madrid, Spain; 6 Department of Rheumatology, Hospital Clínico San Carlos, Madrid, Spain; 7 Department of Rheumatology, Hospital Universitario de Bellvitge-IDIBELL, L'Hospitalet de Llobregat, Barcelona, Spain; 8 Department of Rheumatology, Hospital Universitario Marqués de Valdecilla, IFIMAV, Santander, Spain; 9 Department of Rheumatology, Oslo University Hospital Rikshospitalet, Oslo, Norway; 10 Department of Rheumatology, Hospital of Southern Norway Trust, Kristiansand, Norway; 11 Department of Internal Medicine, Division of Nephrology, Robert-Bosch-Hospital, Stuttgart, Germany; 12 Department of Clinical Immunology and Rheumatology, University of Luebeck, Bad Bramstedt, Germany; 13 Hannover Medical School, Hannover, Germany; 14 Referral Center for Systemic Autoimmune Diseases, Fondazione IRCCS Ca'Granda Ospedale Maggiore Policlinico di Milano, Milan, Italy; 15 Department of Medicine, Università degli Studi di Verona, Verona, Italy; 16 Department of Internal Medicine, Academic Unit of Clinical Rheumatology, University of Genova, Genova, Italy; 17 Department of Clinical Medicine, Nephrology and Health Sciences, University Hospital of Parma, Parma, Italy; 18 Unità Operativa di Reumatologia, Azienda Ospedaliera ASMN, Istituto di Ricovero e Cura a Carattere Scientifico, Reggio Emilia, Italy; IFOM, Fondazione Istituto FIRC di Oncologia Molecolare, Italy

## Abstract

**Introduction:**

Increased expression of IL-33 and its receptor ST2, encoded by the *IL1RL1* gene, has been detected in the inflamed arteries of giant cell arteritis (GCA) patients. The aim of the present study was to investigate for the first time the potential influence of the *IL33* and *IL1RL1 loci* on GCA predisposition.

**Methods:**

A total of 1,363 biopsy-proven GCA patients and 3,908 healthy controls from four European cohorts (Spain, Italy, Germany and Norway) were combined in a meta-analysis. Six genetic variants: rs3939286, rs7025417 and rs7044343, within the *IL33* gene, and rs2058660, rs2310173 and rs13015714, within the *IL1RL1* gene, previously associated with immune-related diseases, were genotyped using predesigned TaqMan assays.

**Results:**

A consistent association between the rs7025417 polymorphism and GCA was evident in the overall meta-analysis, under both allele (P_MH_ = 0.041, OR = 0.88, CI 95% 0.78–0.99) and recessive (P_MH_ = 3.40E-03, OR = 0.53, CI 95% 0.35–0.80) models. No statistically significant differences between allele or genotype frequencies for the other *IL33* and *IL1RL1* genetic variants were detected in this pooled analysis.

**Conclusions:**

Our results clearly evidenced the implication of the *IL33* rs7025417 polymorphism in the genetic network underlying GCA.

## Introduction

Giant cell arteritis (GCA) is a chronic granulomatous vasculitis of large and medium size blood vessels which affects predominantly women and people generally older than 50 years [1].

Although the mechanisms triggering this pathology remain unclear, both immune and genetic factors appear to participate in its pathogenesis. The inflammatory process occurring in GCA is mainly driven by Th1 and Th17 lymphocytes and macrophages [2]. Additionally, endothelial cells also play an important role in the induction and perpetuation of the inflammation by promoting neoangiogenesis [3]. Regarding the genetic component of this vasculitis, besides HLA, a few risk factors have been consistently associated with GCA so far [4], [5] and, therefore, most of the genetic contribution to this disorder continues unidentified.

IL-33 has recently been described as a novel IL-1 family member with strong immunomodulatory functions. This cytokine, through binding to its receptor ST2 (suppression of tumorigenicity 2), encoded by the interleukin 1 receptor-like 1 (*IL1RL1*) gene, activates mast cells and Th2 lymphocytes, leading to the production of chemokines, pro-inflammatory and Th2-associated cytokines, and increased serum immunoglobulin levels [6]. Additionally, it has been shown that IL-33 acts as an activator of endothelial cells promoting angiogenesis and vascular permeability *in vitro* and *in vivo* [7].

Different studies have supported a pathogenic role of IL-33 axis in autoimmunity [8], [9], [10], [11]. Regarding genetic studies, several polymorphisms within the *IL33* region have been associated with different immune-related conditions. Specifically, rs3939286 was associated with asthma [12], nasal polyposis [13] and inflammatory bowel disease (IBD) [14], rs7025417 was associated with coronary artery disease (CAD) [15], and rs7044343 with rheumatoid arthritis [16]. In addition, three other *IL33* polymorphisms, in high linkage disequilibrium (LD) with rs3939286 (rs928413, rs2381416 and rs1342326), have been related to asthma through genome-wide association studies (GWASs) [17], [18], [19]. On the other hand, a number of *IL1RL1* polymorphisms have also been implicated in autoimmunity. In this line, the rs2058660, rs2310173 and rs13015714 genetic variants were associated with Crohn's disease [20], ulcerative colitis and ankylosing spondylits [21], [22], and, celiac disease and inflammatory bowel disease [14], [23], respectively. Moreover, an association between three tightly linked polymorphisms (rs10197862, rs13408661 and rs3771180) and asthma has also been described in GWASs [18], [24], [25].

Interestingly, an increased expression of IL-33 and ST2 has been detected in the inflamed arteries of GCA patients, mainly in endothelial cells of newly formed vessels, thus suggesting a possible role of IL-33 in the angiogenesis-dependent inflammation in GCA [26].

The aim of the present study was to assess whether genetic variants at *IL33* and *IL1RL1*, previously associated with immune-mediated diseases, are involved in the genetic predisposition to this vasculitis.

## Methods

### Study population

The case-control study included a total of 1,363 biopsy-proven GCA patients and 3,908 unrelated healthy controls, both of European ancestry. First, a discovery cohort, consisting of 894 Spanish GCA cases and 2,047 controls was analyzed. Subsequently, a replication phase was conducted in three independent cohorts from Germany (103 cases and 444 controls), Italy (255 cases and 1,141 controls) and Norway (111 cases and 276 controls). **Table S1** summarizes the main characteristics of the analyzed cohorts. Case/control sets were matched by geographical origin and ethnicity. Informed written consent from all participants and approval from the local ethical committees (Comité de Bioética del Consejo Superior de Investigaciones Científicas, Comitato di Etica e Sperimentazione Farmaci Fondazione IRCCS Ca' Granda-Ospedale Maggiore Policlinico di Milano, Ethic Committee Provinciale of Reggio Emilia, Local Ethics Committees of the Hospital Vall d'Hebron, Hospital Clinic, Hospital de la Princesa, Hospital Clínico San Carlos, Hospital Universitario de Bellvitge, Hospital Universitario Marqués de Valdecilla, Oslo University Hospital, Hospital of Southern Norway Trust, Robert-Bosch-Hospital, University of Luebeck, Hannover Medical School, Università degli Studi di Verona, University of Genova and University Hospital of Parma) were obtained in accordance with the tenets of the Declaration of Helsinki. All patients had a positive temporal artery biopsy (disruption of the internal elastic laminae with infiltration of mononuclear cells into the arterial wall with or without multinucleated giant cells) and fulfilled the 1990 American College of Rheumatology classification criteria [27]. Patients were stratified according to the presence or absence of polymyalgia rheumatica (PMR), visual ischaemic manifestations (VIM; if they experienced transient visual loss including amaurosis fugax, permanent visual loss, or diplopia) and irreversible occlusive disease (IOD; if they had at least one of the following features: permanent visual loss, stroke or occlusive disease in the upper extremities or lower extremities).

### Genotyping methods

The selection of single-nucleotide polymorphisms (SNPs) was based on their position and previous association with several inflammatory conditions. Polymorphisms with minor allele frequencies lower than 10% were not included as power calculations indicated a lack of statistical power to analyze them. Following these criteria, six polymorphisms, three in the *IL33* region (rs3939286, rs7025417 and rs7044343), located in different haplotype blocks of this *locus*, and three in the *IL1RL1* region (rs2058660, rs2310173 and rs13015714), were genotyped using the TaqMan allelic discrimination assay technology on a 7900HT Fast Real-Time PCR System, both from Applied Biosystems (Foster City, California, USA).

### Statistical analysis

**Table S2** shows the overall statistical power of the analysis (http://www.sph.umich.edu/csg/abecasis/CaTS/). Plink (v1.07) (http://pngu.mgh.harvard.edu/purcell/plink/) and StatsDirect v.2.6.6 (StatsDirect Ltd, Cheshire, UK) were used to perform 2×2 contingency tables and χ^2^ test. Odds ratios (OR) and 95% confidence intervals (CI) were obtained according to Woolf's method. P-values of the discovery cohort were corrected by The Benjamini & Hochberg (1995) step-up false discovery rate (FDR) control correction for multiple testing [28]. P-values <0.05 were considered statistically significant. The analysis of the combined data from all populations was performed using Plink and StatsDirect. Breslow–Day (BD) test and Cochran's Q and I^2^ statistics were used to estimate the homogeneity among populations. Pooled analyses were performed by Mantel-Haenszel (MH) test under fixed effects.

## Results

Genotypic frequencies did not deviate from those predicted by Hardy-Weinberg (P>0.01) and the genotype success rate were >95%.

**Table 1** shows the genotype and allelic frequencies of the *IL33* and *IL1RL1* polymorphisms analyzed in the Spanish discovery cohort. In the allele test, none of the studied polymorphisms showed a significant association with GCA, although a trend of association was evident for the *IL33* rs7025417 SNP (P = 0.082, OR = 0.87 [0.75–1.02]). When the recessive model was considered, the frequency of the minor genotype of *IL33* rs7025417 was significantly reduced in patients compared to healthy controls (P = 7.04E-03, OR = 0.46 [0.26–0.81]), even after applying FDR correction (P_FDR_ = 0.042). No significant differences were found between patients with and without specific clinical features, *i*.*e*. PMR, VIM and IOD, (data not shown). Regarding *IL1RL1*, none of the analyzed genetic variants showed association with GCA in the case/control (**Table 1**) or subphenotype analysis (data not shown).

**Table 1:** Genotype and minor allele frequency of the *IL1RL1* and *IL33* analyzed polymorphisms in biopsy-proven GCA patients and controls from Spain.

				Genotype, N (%)				Allele test			Recessive model		
SNP	Locus	1/2	Subgroup (N)	1/1	1/2	2/2	MAF (%)	*P*-value	*P*_FDR_ *	OR [CI 95%]**	*P*-value	*P*_FDR_ *	OR [CI 95%]**
rs2310173	*IL1RL1*	T/G	Controls (n = 1972)	458 (23.23)	958 (48.58)	556 (28.19)	47.52						
			GCA (n = 863)	205 (23.75)	395 (45.77)	263 (30.48)	46.64	0.5434	0.9304	0.97 [0.86–1.08]	0.7594	0.9458	1.03 [0.85–1.24]
rs13015714	*IL1RL1*	G/T	Controls (n = 1944)	138 (7.10)	729 (37.50)	1077 (55.40)	25.85						
			GCA (n = 868)	61 (7.03)	329 (37.90)	478 (55.07)	25.98	0.9178	0.9304	1.01 [0.88–1.15]	0.9458	0.9458	0.98 [0.72–1.35]
rs2058660	*IL1RL1*	G/A	Controls (n = 2005)	141 (7.03)	767 (38.25)	1097 (54.71)	26.16						
			GCA (n = 858)	52 (6.06)	343 (39.98)	463 (53.96)	26.05	0.9304	0.9304	0.99 [0.87–1.13]	0.3425	0.6850	0.85 [0.61–1.19]
rs3939286	*IL33*	T/C	Controls (n = 1983)	190 (9.58)	826 (41.65)	967 (48.76)	30.41						
			GCA (n = 862)	80 (9.28)	362 (42.00)	420 (48.72)	30.28	0.9219	0.9304	0.99 [0.88–1.12]	0.8014	0.9458	0.97 [0.73–1.27]
rs7025417	*IL33*	C/T	Controls (n = 1969)	72 (3.66)	533 (27.07)	1364 (69.27)	17.19						
			GCA (n = 871)	15 (1.72)	237 (27.21)	619 (71.07)	15.33	0.0818	0.4907	0.87 [0.75–1.02]	**7.04E-03**	**0.0423**	0.46 [0.26–0.81]
rs7044343	*IL33*	C/T	Controls (n = 1957)	220 (11.24)	865 (44.20)	872 (44.56)	33.34						
			GCA (n = 864)	114 (13.19)	377 (43.63)	373 (43.17)	35.01	0.2218	0.6655	1.08 [0.96–1.21]	0.1393	0.4180	1.20 [0.94–1.53]

*Benjamini and Hochberg step-up false discovery rate control. ** Odds ratio for the minor allele or genotype.

MAF, minor allele frequency; GCA, giant cell arteritis.

**Figure 1:**
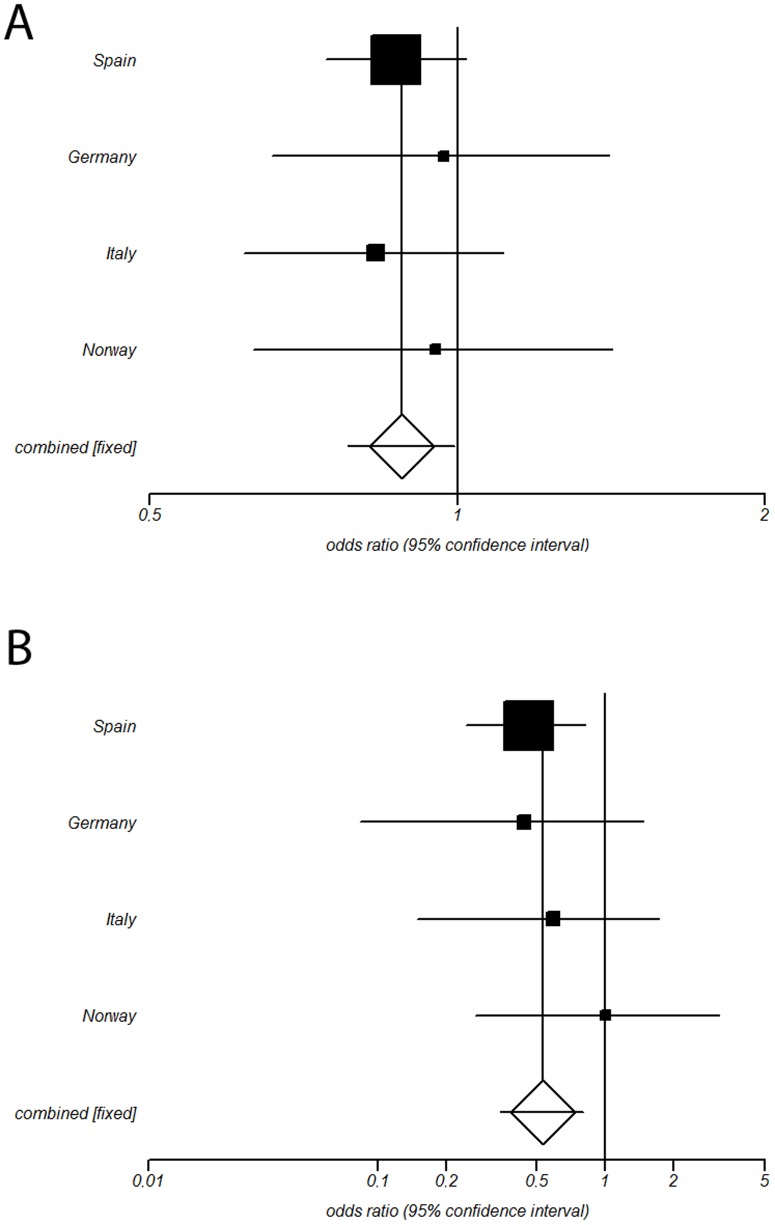
Forest plots showing the odds ratios (OR) and confidence intervals (CI) of the *IL33* rs2075417 genetic variant under allelic (A) and recessive (B) genetic models in the discovery and replication cohorts.

To further examine these findings, the three *IL33* genetic variants were analyzed in three independent case/control sets from Germany, Italy and Norway (**Table 2**). In the Italian set, a clear association between the rs3939286 polymorphism and GCA was found (P = 2.37E-03, OR = 0.70 [0.55–0.88]). No association between *IL33* genetic variants and GCA was evident in the German and Norwegian sets. Subsequently, since no heterogeneity between the ORs from the four cohorts was evident (P_BD_>0.1; Cochran's Q-statistic p>0.1; I^2^ = 0% for rs7025417 and rs7044343; I^2^ = 16% for rs3939286), they were combined in a meta-analysis. As shown in **Table 2**, only the rs7025417 polymorphism showed a consistent association with GCA in both allele (P = 0.041, OR = 0.88 [0.78–0.99], **Figure 1A**) and recessive model (P = 3.40E-03, OR = 0.53 [0.35–0.80], **Figure 1B**). The subphenotype analysis according to the main clinical features of GCA yielded negative results in the pooled analysis (data not shown).

**Table 2:** Replication and pooled analysis of the tested *IL33* genetic variants in European biopsy-proven GCA patients and controls.

				Genotype, N (%)				Allele test		Recessive model	
	SNP	1/2	Subgroup (N)	1/1	1/2	2/2	MAF (%)	*P*-value	OR [CI 95%]*	*P*-value	OR [CI 95%]*
Germany	rs3939286	T/C	Controls (n = 427)	28 (6.56)	147 (34.43)	252 (59.02)	23.77				
			GCA (n = 102)	3 (2.94)	37 (36.27)	62 (60.78)	21.08	0.4135	0.86 [0.59–1.24]	0.1741	0.43 [0.13–1.45]
	rs7025417	C/T	Controls (n = 430)	28 (6.51)	149 (34.65)	253 (58.84)	23.84				
			GCA (n = 101)	3 (2.97)	41 (40.59)	57 (56.44)	23.27	0.8640	0.97 [0.67–1.39]	0.1833	0.44 [0.13–1.48]
	rs7044343	C/T	Controls (n = 436)	67 (15.37)	201 (46.10)	168 (38.53)	38.42				
			GCA (n = 101)	16 (15.84)	51 (50.50)	34 (33.66)	41.09	0.4828	1.12 [0.82–1.53]	0.9054	1.04 [0.57–1.88]
Italy	rs3939286	T/C	Controls (n = 1109)	94 (8.48)	437 (39.40)	578 (52.12)	28.18				
			GCA (n = 251)	13 (5.18)	82 (32.67)	156 (62.15)	21.51	**2.37E-03**	0.70 [0.55–0.88]	0.0830	0.59 [0.32–1.07]
	rs7025417	C/T	Controls (n = 1106)	29 (2.62)	282 (25.50)	795 (71.88)	15.37				
			GCA (n = 255)	4 (1.57)	59 (23.14)	192 (75.29)	13.14	0.2023	0.83 [0.63–1.10]	0.3295	0.59 [0.21–1.70]
	rs7044343	C/T	Controls (n = 1116)	188 (16.85)	520 (46.59)	408 (36.56)	40.14				
			GCA (n = 252)	46 (18.25)	116 (46.03)	90 (35.71)	41.27	0.6415	1.05 [0.86–1.28]	0.5920	1.10 [0.77–1.57]
Norway	rs3939286	T/C	Controls (n = 271)	15 (5.54)	100 (36.90)	156 (57.56)	23.99				
			GCA (n = 108)	6 (5.56)	45 (41.67)	57 (52.78)	26.39	0.4883	1.14 [0.79–1.63]	0.9937	1.00 [0.38–2.66]
	rs7025417	C/T	Controls (n = 267)	12 (4.49)	91 (34.08)	164 (61.42)	21.54				
			GCA (n = 111)	5 (4.50)	36 (32.43)	70 (63.06)	20.72	0.8032	0.95 [0.65–1.40]	0.9965	1.00 [0.34–2.92]
	rs7044343	C/T	Controls (n = 273)	43 (15.75)	135 (49.45)	95 (34.80)	40.48				
			GCA (n = 108)	14 (12.96)	45 (41.67)	49 (45.37)	33.80	0.0878	0.75 [0.54–1.04]	0.4924	0.80 [0.42–1.53]
Meta-analysis	rs3939286	T/C	Controls (n = 3790)	327 (8.63)	1510 (39.84)	1953 (51.53)	28.55				
			GCA (n = 1323)	102 (7.71)	526 (39.76)	695 (52.53)	27.59	0.1358	0.93 [0.84–1.02]	0.2036	0.85 [0.67–1.08]
	rs7025417	C/T	Controls (n = 3772)	141 (3.74)	1055 (27.97)	2576 (68.29)	17.72				
			GCA (n = 1338)	27 (2.02)	373 (27.88)	938 (70.10)	15.96	**0.0408**	0.88 [0.78–0.99]	**3.40E-03**	0.53 [0.35–0.80]
	rs7044343	C/T	Controls (n = 3782)	518 (13.70)	1721 (45.51)	1543 (40.80)	36.45				
			GCA (n = 1325)	190 (14.34)	589 (44.45)	546 (41.21)	36.57	0.3747	1.04 [0.95–1.14]	0.2447	1.12 [0.93–1.34]

*Odds ratio for the minor allele or genotype.

MAF, minor allele frequency; GCA, giant cell arteritis.

## Discussion

In the present study, we identified for the first time an association between the *IL33* gene and GCA through a large meta-analysis of four European cohorts. The minor allele (C) and genotype (CC) of the rs7025417 genetic variant showed a protective effect, consistently with that described in a previous study performed in CAD patients [15]. Interestingly, this polymorphism seems to produce an altered regulation of the *IL33* gene expression [15]. In addition, the rs7025417 risk allele (T) correlates with an increase of the IL-33 plasma level in CAD [15]. Our findings would suggest that the rs7025417 genetic variant might influence the development of GCA by regulating the expression of IL-33 [26].

Although the combined analysis of the four cohorts evidenced that only one of the three *IL33* tested polymorphisms, rs7025417, was associated with GCA (probably due to the larger sample size of the Spanish set), another different SNP, rs3939286, showed a clear association in the Italian cohort. Both *IL33* genetic variants, rs7025417 and rs3939286, have been previously associated with autoimmune diseases [12], [13], [14], [15] and they appear to be regulatory DNA elements according to the public database RegulomeDB [29], but with minimal evidence of being located in a functional region (scores 5 and 6, for rs7025417 and rs3939286, respectively). As for rs7025417, the minor allele of the rs3939286 conferred protection to GCA, which is the opposite effect to that previously reported in asthma, nasal polyposis and IBD [12], [13], [14]. This could be indicating a different molecular effect of this SNP in the pathogenesis of GCA than in other autoimmune diseases. In addition, both SNPs are located in different haplotype blocks and present a low LD (D′ = 0.13 and r^2^ = 0.01), which suggests that they may represent independent signals. Nevertheless, the fact that different *IL33* polymorphisms were associated with GCA in different populations may indicate that none of them is the real causal variant of the association but genetic markers in linkage disequilibrium with it. Small changes in the LD pattern between populations might result in different SNPs being the most associated with the disease in different cohorts.

In spite of an increased expression of the ST2 receptor was detected in the inflamed arteries of GCA patients [26], none of the tested *IL1RL1* polymorphisms showed association with this vasculitis. Taking into account that the statistical power of our analysis was high enough to detect a possible weak signal (power >80% to detect an OR >1.20 in the discovery cohort), our data rule out an important role of the analyzed *IL1RL1* genetic variants in GCA.

Angiogenesis has been proposed as one of the main mechanisms influencing the progression of GCA [30]. Interplay between inflammation and angiogenesis is mainly mediated by cytokines, chemokines, and growth factors, which also induce increase vascular permeability. In recent years, genetic studies have supported this crucial role of angiogenesis in GCA. In this line, variations within the vascular endothelial growth factor (*VEGF*) gene, encoding the best known angiogenic factor, have been associated with this vasculitis [4]. Like VEGF, IL-33 also exerts its angiogenic and vasopermeability activities in a nitric oxide (NO)-dependent manner [7]. Interestingly, a role for two genes encoding NO synthases (*NOS2A*, cytokine-inducible, and *NOS3*, endothelial) has also been demonstrated in GCA [4].

In conclusion, our results clearly indicate a role of the *IL33* rs7025417 polymorphism as a novel genetic risk factor contributing to the GCA susceptibility. The effect of additional and unexplored *IL33* and *IL1RL1* genetic variants in GCA susceptibility cannot be discarded.

## Supporting Information

Table S1 **Main clinical features of the giant cell arteritis patients included in the study.**
(DOCX)

Table S2 **Overall statistical power of the study for each analyzed** ***IL1RL1*** **and** ***IL33*** **genetic variant at the 5% significance level.**
(DOCX)

File S1 **Other members of Spanish GCA Consortium contributing samples and clinical data to this analysis.**
(DOCX)
