# Supplementary material for: A Candidate Gene Approach Identifies an IL33 Genetic Variant as a Novel Genetic Risk Factor for GCA
Source: PLoS One. 2014 Nov 19;9(11):e113476. doi: 10.1371/journal.pone.0113476 (PMC4237421; doi:10.1371/journal.pone.0113476)
Supplement: Table S1 — Main clinical features of the giant cell arteritis patients included in the study. (DOCX) [file pone.0113476.s001.docx]

**Table S1.** Main clinical features of the giant cell arteritis patients included in the study.

|  | **Number (%)** | | | |
| --- | --- | --- | --- | --- |
| **Feature** | **Spain** | **Germany**^a^ | **Italy** | **Norway** |
| Age at diagnosis, years, median (IQR) | 75 (71-80) | 69 (64-76) | 74 (68-79) | 71 (65-77) |
| Female | 622 (69.6) | 72 (76.4) | 211 (81.2) | 71 (83.5) |
| Headache | 725 (84.3) | 41 (74.5) | 180 (82.2) | 73 (88.0) |
| Abnormal temporal artery on examination | 580 (70.1) | 20 (40.0) | 144 (68.6) | 38 (69.1)^a^ |
| Polymyalgia rheumatica | 388 (44.4) | 21 (38.2) | 99 (45.2) | 32 (37.6) |
| Jaw claudication | 392 (45.2) | 15 (27.3) | 101 (47.0)^b^ | 11 (50.0)^C^ |
| Arm-leg claudication | 52 (6.0) | 6 (10.9) | 7 (6.4) | 1 (4.5)^C^ |
| Visual ischemic manifestations* | 266 (30.5) | 11 (20.0) | 80 (36.5) | 23 (28.4) |
| Permanent visual loss | 137 (16.0) | 1 (1.8) | 45 (20.5) | 8 (9.9) |
| Stroke | 47 (5.4) | 1 (2.0) | 4 (1.8) | 2 (2.5) |
| Severe ischemic manifestations** | 406 (49.8) | 1 (1.8) | 133 (61.0) | 21 (25.9) |
| Oclussive Vascular Disease*** | 159 (18.7) | 2 (3.8) | 46 (21.3) | 6 (7.4) |

IQR, interquartile range.

*Transient visual loss including amaurosis fugax, permanent visual loss, or diplopia.

**At least one of the following features: visual manifestations, cerebrovascular accidents (stroke and/or transient ischemic attacks), jaw claudication, or limb claudication.

***Whether permanent visual loss or stroke is present.

^a^ Data from 50% of the patients; ^b^ Data from 42% of the patients; ^C^ Data from 20% of the patients
