# Supplementary material for: A Candidate Gene Approach Identifies an IL33 Genetic Variant as a Novel Genetic Risk Factor for GCA
Source: PLoS One. 2014 Nov 19;9(11):e113476. doi: 10.1371/journal.pone.0113476 (PMC4237421; doi:10.1371/journal.pone.0113476)
Supplement: Table S2 — Overall statistical power of the study for each analyzed IL1RL1 and IL33 genetic variant at the 5% significance level. (DOCX) [file pone.0113476.s002.docx]

**Table S2**. Overall statistical power of the study for each analyzed *IL1RL1* and *IL33* genetic variant at the 5% significance level.

|  | **SNP** | **OR=1.1** | **OR=1.2** | **OR=1.3** |
| --- | --- | --- | --- | --- |
| ***IL1RL1*** | rs2310173 | 0.39 | 0.90 | 1.00 |
|  | rs13015714 | 0.31 | 0.81 | 0.99 |
|  | rs2058660 | 0.32 | 0.82 | 0.99 |
| ***IL33*** | rs3939286 | 0.34 | 0.85 | 0.99 |
|  | rs7025417 | 0.24 | 0.70 | 0.95 |
|  | rs7044343 | 0.35 | 0.87 | 0.99 |
