# Supplementary material for: A Candidate Gene Approach Identifies an IL33 Genetic Variant as a Novel Genetic Risk Factor for GCA
Source: PLoS One. 2014 Nov 19;9(11):e113476. doi: 10.1371/journal.pone.0113476 (PMC4237421; doi:10.1371/journal.pone.0113476)
Supplement: File S1 — Other members of Spanish GCA Consortium contributing samples and clinical data to this analysis. (DOCX) [file pone.0113476.s003.docx]

**File S1**

Other members of Spanish GCA Consortium contributing samples and clinical data to

this analysis:

**José A Miranda-Filloy,** Department of Rheumatology, Hospital Xeral-Calde, Lugo; **María Encarnación Fernández-Contreras**, Department of Pathology, Hospital de la Princesa, IIS-Princesa, Madrid; **Carmen Gómez-Vaquero**, Department of Rheumatology, Hospital Universitario de Bellvitge-IDIBELL, L'Hospitalet de Llobregat, Barcelona; **Eugenio de Miguel,** Department of Rheumatology, Hospital Universitario La Paz, Madrid; **Bernardo Sopeña**, Department of Internal Medicine, Complejo Hospitalario Universitario de Vigo; **María Jesús García-Villanueva**, Department of Rheumatology, Hospital Ramón y Cajal, Madrid; **Begoña Marí-Alfonso**, Department of Internal Medicine, Corporació Sanitaria Parc Taulí, Instituto Universitario Parc Taulí, UAB, Sabadell, Barcelona; **Ainhoa Unzurrunzaga**, Department of Internal Medicine, Hospital de Galdakano, Vizcaya; **Julio Sánchez-Martín**, Department of Rheumatology, Hospital Universitario 12 de Octubre, Madrid; **Ana Hidalgo-Conde**, Department of Internal Medicine, Hospital Universitario Virgen de la Victoria, Málaga; **Patricia Fanlo Mateo**, Department of Internal Medicine, Hospital Virgen del Camino, Pamplona; **Sergio Prieto-González** and **Marc Corbera-Bellalta**, Vasculitis Research Unit, Department of Autoimmune and Systemic Diseases, Hospital Clinic, University of Barcelona, Centre de Recerca Biomèdica Cellex (IDIBAPS), Barcelona; **Benjamín Fernández-Gutiérrez** and **Inmaculada C Morado**, Department of Rheumatology, Hospital Clínico San Carlos, Madrid; **Elena Grau** and **José Andrés Román**, Department of Rheumatology, Hospital Universitario y Politécnico La Fe, Valencia; **José Bernardino Díaz López**, **Aleida Martínez Zapico** and **Luis Caminal-Montero**, Department of Internal Medicine, Hospital Central de Asturias, Oviedo; **Antonio Fernández-Nebro** and **María Carmen Ordóñez Cañizares**, Rheumatology Department, Hospital Carlos Haya, Málaga; **Raquel Rios**, Department of Internal Medicine, Hospital Clínico San Cecilio, Granada; **César Magro** and **Enrique Raya**, Department of Rheumatology, Hospital Clínico Universitario San Cecilio, Granada; **Jordi Monfort** and **Laura Tío**, Department of Rheumatology, Grup de recerca cel•lular en inflamació i cartílag. IMIM (Institut de Recerca Hospital del Mar), Barcelona; **Francisco Javier López-Longo** and **Lina Martínez**, Department of Rheumatology, Hospital General Universitario Gregorio Marañón, Madrid; **Luis Sáez-Comet** and **Mercedes Pérez-Conesa**, Department of Internal Medicine, Hospital Universitario Miguel Servet, Zaragoza.
